# Culture-sensitive psychotraumatology

**DOI:** 10.3402/ejpt.v7.31179

**Published:** 2016-07-28

**Authors:** Ulrich Schnyder, Richard A. Bryant, Anke Ehlers, Edna B. Foa, Aram Hasan, Gladys Mwiti, Christian H. Kristensen, Frank Neuner, Misari Oe, William Yule

**Affiliations:** 1 Department of Psychiatry and Psychotherapy, University Hospital, University of Zurich, Zurich, Switzerland; 2 School of Psychology, University of New South Wales, Sydney, NSW, Australia; 3 Department of Experimental Psychology and Oxford Cognitive health NIHR Clinical Research Facility, University of Oxford, Oxford, UK; 4 Department of Psychiatry, Center for the Treatment and Study of Anxiety, University of Pennsylvania, Philadelphia, PA, USA; 5 Centrum'45, Oegstgeest, The Netherlands; 6 Oasis Africa Center for Transformational Psychology & Trauma, Nairobi, Kenya; 7 Centre of Studies and Research in Traumatic Stress, Pontifical Catholic University of Rio Grande do Sul, Porto Alegre, Brazil; 8 Department of Clinical Psychology and Psychotherapy, Bielefeld University, Bielefeld, Germany; 9 Department of Neuropsychiatry, Kurume University School of Medicine, Fukuoka, Japan; 10 Department of Applied Child Psychology, King's College, London, UK

**Keywords:** Culture, psychotraumatology, stigma, low and middle income countries, dissemination, evidence-based treatments

## Abstract

**Background:**

Although there is some evidence of the posttraumatic stress disorder (PTSD) construct's cross cultural validity, trauma-related disorders may vary across cultures, and the same may be true for treatments that address such conditions. Experienced therapists tailor psychotherapy to each patient's particular situation, to the nature of the patient's psychopathology, to the stage of therapy, and so on. In addition, culture-sensitive psychotherapists try to understand how culture enhances the meaning of their patient's life history, the cultural components of their illness and help-seeking behaviors, as well as their expectations with regard to treatment. We cannot take for granted that all treatment-seeking trauma survivors speak our language or share our cultural values. Therefore, we need to increase our cultural competencies.

**Methods:**

The authors of this article are clinicians and/or researchers from across the globe, working with trauma survivors in various settings. Each author focused on one or more specific cultural aspects of working with trauma survivors and highlighted the following aspects.

**Results:**

As a result of culture-specific individual and collective meanings linked to trauma and trauma-related disorders survivors may be exposed to (self-)stigma in the aftermath of trauma. Patients who are reluctant to talk about their traumatic experiences may instead be willing to write or use other ways of accessing the painful memories such as drawing. In other cultures, community and family cohesion are crucial elements of recovery. While awareness of culture-specific aspects is important, we also need to beware of premature cultural stereotyping. When disseminating empirically supported psychotherapies for PTSD across cultures, a number of additional challenges need to be taken into account: many low and middle income countries have very limited resources available and suffer from a poor health infrastructure.

**Conclusions:**

In summary, culture-sensitive psychotraumatology means assuming an empathic and non-judgmental attitude, trying to understand each individual's cultural background.

**Highlights of the article:**

- Cultural sensitivity is required to successfully treat trauma survivors.
- Empirically supported treatments may need to be modified depending on the patient’s cultural background.
- In addition to talking, patients may write or use other ways of accessing the traumatic memories such as painting, dancing, or singing.
- In low and middle income countries, dissemination of evidence-based treatments is frequently impeded by limited resources and poor health infrastructure.
- Beware of cultural stereotyping!

The field of psychotraumatology has developed a number of evidence-based psychotherapies for the treatment of trauma-related disorders (Bisson, Roberts, Andrew, Cooper, & Lewis, 2013; Bradley, Greene, Russ, Dutra, & Westen, 2005; Schnyder & Cloitre, 2015; Watts et al., 2013). These approaches differ in various ways including the duration and number of sessions, as well as the number and diversity of interventions. However, they also have many commonalities, such as psychoeducation, imaginal exposure, cognitive restructuring, or meaning making (Schnyder et al., 2015). Although there is some evidence of the posttraumatic stress disorder (PTSD) construct's cross-cultural validity, trauma-related disorders may vary across cultures (Hinton & Lewis-Fernández, 2011), and the same may be true for treatments of these posttraumatic conditions. There is also increasing evidence of cultural differences influencing not only psychological mechanisms but trauma-related neural processes and substrates as well (Liddell & Jobson, 2016).

Wen-Shing Tseng, the founding president of the World Association of Cultural Psychiatry, defined culture as a dynamic concept referring to a set of beliefs, attitudes, and value systems, which derive from early stages of life through enculturation and become an internal mode of regulating behavior, action, and emotion (Tseng & Streltzer, 2001). Thus, culture is not static but changes continuously across generations and responds to ever-changing environmental demands. Furthermore, culture in Tseng's sense is specific for each individual and therefore much more important than ethnicity or race. Experienced therapists usually tailor psychotherapy to each patient's particular situation, the nature of the patient's psychopathology, the stage of progress in the course of therapy, as well as a range of other factors. Treatment could be even more effective, however, if the cultural dimensions were to be incorporated. Culture-sensitive psychotherapy involves trying to understand how culture enhances the meaning of the patient's life history, the cultural components of a patient's illness and help-seeking behaviors, as well as the patient's expectations with regard to treatment.

Trauma is a global issue (Schnyder, 2013). Our traumatized patients come from all over the world. We cannot take for granted that they all speak our language or share our cultural values. Therefore, we need to increase our cultural competencies. Being sensitive to cultural issues has become a sine qua non for being a good therapist. On the one hand, taking into account the cultural dimension adds yet one more challenge to our already demanding profession. On the other hand, it also enriches our work, providing us with opportunities to learn how diverse human beings are and how different a phenomenon such as a flashback or a certain aspect of a traumatic experience can be understood and interpreted depending on the patient's and their therapist's cultural backgrounds.

This paper is based on a panel discussion that was held at the annual meeting of the International Society for Traumatic Stress Studies in New Orleans, USA, in November 2015. A number of additional international experts in the field (Frank Neuner, Misari Oe, William Yule and Richard Bryant) were invited to write their comments afterwards. The authors of this article are clinicians and/or researchers from across the globe, working with trauma survivors in various settings. We all share the belief that a better understanding of the cultural dimension of psychotraumatology will make us better therapists. Each author will focus on one or more specific cultural aspects of working with trauma survivors. The article will conclude with a summary of what we can possibly learn from taking a cultural viewpoint, how evidence-based treatments need to be adjusted when treating patients from various cultural backgrounds, and also in which way new developments in psychotherapies for PTSD and other trauma-related disorders may possibly be informed by these cultural elements.

## Anke Ehlers, University of Oxford, UK: cultural beliefs influence personal meanings

Cultural beliefs may influence an individual's personal meanings of trauma and his or her attempts to come to terms with trauma memories in helpful and unhelpful ways. The meanings linked to PTSD show cultural variations: in individualistic cultures appraisals about a vulnerable or inadequate self are common; in collectivistic cultures appraisals about social functioning or evaluation by others. Cultural beliefs may also influence the reactions of significant others and the community and can thus facilitate or impede recovery from trauma. Our National Health Service outpatient clinic in South London, UK, serves a population from very diverse cultural backgrounds (Ehlers et al., 2013). Over the years, we have learned a lot from our patients about different cultural beliefs and how to adapt to treatment accordingly. Cognitive therapy for PTSD assumes that there are universal psychological mechanisms that promote recovery from PTSD and aims to change idiosyncratic appraisals that induce a sense of current threat, unhelpful ways of coping with trauma memories, and the nature of the trauma memory. Treatment is tailored to the individual's beliefs, including his or her cultural beliefs. I will give a few examples:

If possible, cognitive therapy aims to change the threatening appraisals of the trauma within the patient's belief system. The aim is to see the traumatic meanings in a new light, by considering a wider range of information than available during the trauma. Obtaining relevant information from respected individuals in the community can be very helpful. For example, a man who was recognized as a prophet in his community interpreted intrusive images of his own funeral as a sign that he was going to die soon. A discussion with his church elders about how prophecies can be distinguished from other mental images helped him realize that his images were a result of worrying about death rather than a prophecy.

Sometimes a reflection on rigidly held cultural beliefs is needed to overcome the effects of trauma, ideally supported by people from the patient's cultural background who have a more nuanced view. Video testimonies of other trauma survivors and surveys can be very helpful. For example, in some cultures, it is believed that rape victims bring shame on their families. A rape survivor was told that she no longer belonged to her family and was sent to live abroad. She lived in dread that others could find out about the rape. In therapy, she reflected with her therapist on cultural values. She realized that shared beliefs in any culture are relative, have advantages and disadvantages, and that they change over time. She realized that not all cultures judge rape survivors in this way, and that even within her culture, not everyone agreed. A survey conducted by her therapist showed that people expressed considerable compassion. This enabled her to seek support from other women from her cultural background who were critical of the cultural beliefs about rape survivors.

Engagement with the trauma memory is an essential part of treatment. Patients who feel ashamed about the trauma may initially be uncomfortable talking about it but may be willing to write in private about the trauma and its meaning, to communicate remotely with the therapist (e.g., via internet-delivered treatment), or use other ways of accessing the painful memories such as drawing, music, working with physical symptoms connected to the trauma, or visiting places that evoke memories.

Rebuilding social activities (e.g., participation in shared activities or simple activities such as cooking a traditional meal) is an important element of treatment, as many trauma survivors feel disconnected from others or their culture, or believe that they have failed in their social roles. Participation in cultural practices can also help overcome threatening meanings, for example, cultural practices after the death of a significant other may help the patient feel connected to others and realize that the deceased is no longer suffering.

## Edna B. Foa, University of Pennsylvania, USA: disseminating treatments across cultures

In the context of treatment, the issue of cross-cultural diversity is particularly pertinent when we aim to disseminate treatments that were developed in one culture in another culture. In the process of doing so, questions arise about the need to modify treatments to increase their effectiveness in the target culture.

Prolonged exposure therapy (PE) was developed in Philadelphia, USA, a city with diverse ethnic groups such as African Americans, Latinos, Asians, and Caucasians. Our outcome studies included participants from all these groups with no outcome differences among them. Similarly, in non-Western cultures such as Japan and Israel, PE yielded the same effectiveness. But these cultures have access to Western cultures such that they influence one another and share some, although not all, basic assumptions. These common aspects allow successful dissemination of PE and other psychosocial treatments without a need to modify them in major ways.

In developing countries, we cannot assume shared concepts about mental health problems, shared knowledge about the psychotherapy or medication that alleviate these problems, and shared views of how to cure mental problems. Here, we need to examine similarities and differences between trauma-related symptoms in order to determine what symptoms to target. Such an examination would inform us whether the targets of our interventions are different or similar, and to what extent we should modify our interventions or develop new ones.

It stands to reason that after a traumatic event (or more often multiple events), an individual would develop a perception of the world as a dangerous place and will resort to avoidance and safety behaviors; and that this perception is invariant across cultures. If so, in vivo exposure to relatively safe situations is likely to help disconfirm and change this perception. But what if the world is really entirely dangerous for individuals with PTSD? This question arises sometimes with regard to patients in Western societies, but then we need to ascertain that the patient is in a relatively safe environment before we exercise in vivo exposure. This may not be possible in other countries such as Brazil (see Christian Kristensen's contribution further down). What then? One way is to institute in vivo exposure in larger groups rather than individually, as it is usually done. And how about the repeated finding that participants in our studies view themselves as entirely incompetent? The assumption that this cognition is invariant across cultures is less intuitive, and we may compromise the outcome of PE when we focus on this negative cognition if it is not an essential component of PTSD in a given target culture. In the short run, studying negative PTSD-related cognitions to assure their relevance in developing countries before disseminating treatments to them is neither feasible nor ethical. But, we can gain insight about the nature of PTSD in developing countries through focused conversations with patients to whom we deliver effective treatments and modify them if necessary as part of the dissemination process.

In this section, I have discussed only some considerations in the decision of when and how to modify existing treatments across cultures. Other aspects of dissemination such as how to adapt training programs and who will deliver treatment are only few of the many questions in this important journey.

## Aram Hasan, Centrum'45, The Netherlands: treating traumatized refugees

The treatment of refugees who are suffering from PTSD is a very complex situation for both patient and therapist. The great majority of traumatized refugees do not receive trauma-focused therapy, largely because cultural differences with regard to, for example, the way of establishing and maintaining a therapeutic relationship or basic ethical values such as trustworthiness and discreetness, are not taken into account sufficiently (Knipscheer & Kleber, 2012). Moreover, most therapists focus on stabilization, psychosocial and community support (Knipscheer & Hasan, 2013), which is not always sufficient to help patients effectively. Also, therapists often move on to exposure therapy too soon or too late. There may be several additional impediments, such as lack of knowledge, incorrect diagnosis, or lack of motivation on the side of the patient and/or the therapist. Over time, post-migration difficulties may even increase and complex PTSD may develop, making trauma-focused therapy an even greater challenge.

Each phase of the treatment is essential for a successful treatment. In the first phase, psychoeducation about PTSD and related complexities is very important. It helps the patients and their family or friends to make connections between the trauma and symptoms (Gersons, Meewisse, Nijdam, & Olff, 2011). It also contributes to a better understanding of their everyday functioning. This helps the patients and their supporters to better articulate their request for help and together with therapists set realistic goals. These objectives are set in an intercultural context. Another extremely important aspect is the establishment of trust so that the refugee feels being taken seriously and understood.

Also, in the trauma-focused phase of therapy, cultural differences should be taken into account. Many patients cannot, or do not dare to write, or are not used to talk about their feelings. In these patients, making use of drawings in the context of trauma-focused therapy can be extremely helpful. Through drawing, traumatized patients are challenged to talk not only about their past but also about their current problems, for instance about their relationship problems they are often ashamed of (Hasan, 2015).

A 51-year-old divorced refugee from Iraq with adolescent daughters had survived as a veteran the very traumatizing war during 8 years between Iraq and Iran and the Gulf war. In 2000, he fled with his family to the Netherlands. He lost his work after a conflict when he became aggressive and threatened his boss with a knife at his throat. When the financial, psychological, and social problems became severe, he was involuntarily hospitalized and then referred to a treatment center for survivors of torture. After two years of different therapies with various therapists without any result, he found a therapist who spoke the same language and had the same family-oriented cultural background. With much psychoeducation about traumatization, he started to make a book of his life and experiences, drawing and writing about his war traumas. This made it possible for him to experience his emotions of anger, sadness, shame, and helplessness. The most difficult for him was to be honest regarding the disappointments, shame, and pain he felt in the relationship with his ex-wife. Finally, the patient accepted his ex-wife's decision to divorce him and was ready to talk about his trauma and engage in trauma-focused treatment. Later, he said that he had never thought he would be able to go through his painful past and all his disappointments. He became happy and proud of himself again, especially after the relationship with his daughters had improved.

## Gladys Mwiti, Oasis Africa, Kenya: community and family cohesion

The call for cultural competency or sensitivity in psychotherapy has increased over the years (Whaley & Davis, 2007). Yet, what is cultural competency? Often, the understanding is that psychotherapists need to be aware of cultural differences between themselves and minority populations and so exercise competency in handling the variances in meanings and approaches to healing modalities. Without cultural sensitivity, psychotherapy is reportedly underutilized, and people terminate services prematurely (Pole, Gone, & Kulkarni, 2008). The reason could be that the treatment does not resonate or connect with the realities of psychological needs.

Currently, most of the evidence-based psychotherapies are derived from Western theories of psychology, while psychologies of developing nations are mainly oral, under-researched, and under-published. What happens if a therapist practices where such populations are the majority? How would we then define cultural sensitivity and what does cultural competency look like? Whether psychotherapy is among minority or majority populations, the secret of uptake is the level of the therapist's emotional connection through respect of the client's culture. This sensitivity is portrayed by the therapist's ability to humbly ask questions, listen, learn, and validate the client's perceptions. Such a therapist will refrain from shaping the client's acuities as per pre-determined theoretical understanding of diagnoses and psychotherapies. Can the therapist be secure enough to allow the client to lead the healing process? Psychotherapy is not a fixed set of steps that can be *cut and pasted* on each client irrespective of individual differences: age, gender, culture, education, and other. Psychotherapy is informed by the principles that govern human behavior and recovery but is shaped by each client and the differences they bring to therapy. This means that capability and efficacy in cultural competency are informed by the client's ethnicity or racial group (Sue, Zane, Hall, & Berger, 2009).

A 25-year-old young woman works for an IT company in Nairobi. She is Kisii by tribe, one of the 42 Kenyan ethnic groups, each with their own different indigenous cultures. These cultures dictate healing methodologies, practices, and rituals that govern mourning especially following traumatic death. The Kisii people live in the western part of Kenya, where this young woman was born and went to school. Later, she joined her 40-year-old sister in Nairobi to attend college. Early this year, coming home from the garden, her mother was struck dead by lightning as she sheltered under a tree during a rainstorm. Kisii culture dictates that after such a death, the deceased should be buried at the very spot where she died. When the young woman received the news of her mother's death, she was too traumatized to travel home for the burial. However, she could not imagine that her mother would have to be buried away from home and the location where she had died. This ritual was discussed in therapy as the patient's distress decreased over the weeks of therapy. The family awaited her coming home before completing funeral rites. Her fiancé was supposed to preside over the burial and so the couple had to postpone their wedding. Finally, when the patient was able to travel home for the burial ceremony, she joined the community and together with her fiancé performed the ritual burial. This brought closure for her and enhanced her recovery from her loss and grief. Participating in this ritual enriched closeness with the family and community.

## Christian Kristensen, Pontifical Catholic University of Rio Grande do Sul, Porto Alegre, Brazil: ongoing violence and impunity

Interpersonal violence (including both community violence, and family and intimate partner violence) in Brazil is high. Brazil had one of the largest estimated rates of homicide in the world, with 32.4 per 100,000 population in 2012 (WHO, 2014), and the highest years of life lost to violence out of any WHO member state (Murray, Cerqueira, & Kahn, 2013). The main victims of domestic violence are poor black women and children (Reichenheim et al., 2011), whereas victims and perpetrators of community violence are most likely to be young, male, and black (Murray et al., 2013). The non-fatal consequences of interpersonal violence result in high collective and individual costs, imposing negative effects in physical and mental health, including PTSD. The estimated prevalence of PTSD in Sao Paulo and Rio de Janeiro is 10.2 and 8.7%, respectively, whereas the prevalence of lifetime exposure to traumatic events is 90% (Ribeiro et al., 2013).

Different types of violence are associated with social determinants such as cultural and social norms, gender issues, poor governance, poor rule of law, unemployment, and inequality (WHO, 2014). According to the Gini Index, Brazil has one of the highest rates of inequality in the world (Murray et al., 2013). Also, widespread corruption and impunity in Brazil favors a culture of permissiveness that nurtures violence (Reichenheim et al., 2011; Waiselfisz, 2010). For instance, an impunity index was the most important predictor of homicide rate in a state-level analysis in Brazil (Nadanovsky, Celeste, Wilson, & Daly, 2009).

The elevated rates of interpersonal and collective violence (especially in the form of organized violent crime) not only are related to the prevalence of traumatic stressors for Brazilians but also can inflict a sense of current and permanent threat that has negative consequences in the treatment of PTSD. It is not rare, in the course of treatment, to see our patients being exposed to additional trauma, with the potential to intensify negative appraisals of the traumatic event, about the world/others and about the self. This continued exposure indicates the need to focus on interventions that booster social support and community bonds as a way of promoting resilience. Beliefs about uncertainty, safety, and danger are challenging themes and should be addressed in cognitive restructuring. Impunity, on the other hand, not only contributes to the occurrence of violence, but has the potential to interfere with psychotherapeutic efforts, in groups or individually. In Brazil, the insufficient responses of the public security forces and the justice system (Reichenheim et al., 2011), and the institutional tolerance to corruption, help to promote impunity which can, in turn, leave trauma victims to increased feelings of helplessness or anger, associated with ruminations about injustices or fantasies of revenge. These issues should also be the focus of cognitive restructuring, and therapeutic efforts should be made in order to help the individual to give meaning to suffering and, hopefully, be part of social change (e.g., joining victims associations, advocacy groups, and community or neighborhood associations).

## Frank Neuner, Bielefeld University, Germany: don't fall for the clichés

A 53-year-old widow had lost her husband in a flood that had destroyed her house and much of her property. Although she was severely traumatized and presented with almost all symptoms of PTSD, she was hesitating to accept any offer for treatment. Psychotherapy was no concept for her as she relied on traditional and religious ways of healing, including the use of local herbal extracts. Her interpretation of the incident was entirely religious, and she felt deeply ashamed as she perceived that various moral transgressions of herself as well as her community were responsible for the incident.

A 30-year-old engineer had been traumatized in a car accident. The detailed exploration of his trauma history revealed a long history of child abuse and various forms of maltreatment that preceded the accident, including severe physical abuse and emotional neglect by both of his parents. He presented with a textbook version of PTSD and depression and lived far below his intellectual possibilities. However, he felt that his severe pain could only be adequately treated by modern medicine. He kept visiting nurses and physical doctors, stayed in hospitals and abused various drugs such as pain killers and benzodiazepines.

These two cases illustrate how culture may shape the meaning of traumatic events as well as the interpretation of trauma symptoms. However, contrary to cultural clichés, the lady stems from a large city in Germany, whereas the gentleman is Southern Sudanese and lives in one of the most remote refugee camps in Uganda. These two case vignettes illustrate that it is necessary to understand the individual context of upbringing to recognize the cultural background of a person. In terms of their attitudes and beliefs, both patients may be exceptional, as their attitudes related to collectivism, traditionalism, and secularism differ much from the stereotypes as well as the statistical averages of their populations. However, psychotherapy is about dealing with exceptions.

In recent years, I met a large number of patients from various cultural backgrounds as part of my research into the development and evaluation of psychotherapy for trauma patients in resource-poor countries such as Uganda, Rwanda, and Sri Lanka. The main conclusion of my experience is that the rapid change of culture as well as the diversity of attitudes within cultures forbids the premature transfer of stereotypes to the individual. Rather, intercultural skills in psychotherapy means following an empathic and non-judgmental effort to understand each individual's system of belief and attitudes. The lifespan biographic approach of Narrative Exposure Therapy (Schauer, Neuner, & Elbert, 2011) provides a good tool to understand the development of a personality in their social context and to understand their specific reactions to traumatic events. Intercultural sensitive psychotherapy means to embed such evidence-based procedures of trauma treatment, most of all exposure and cognitive interventions, into the culturally determined specific belief system of the individual patient. A carefully individualized psychoeducation is the bridge between the world-view of the patient and the evidence-based tools. However, this approach requires that the evidence base had been gained in culturally sensitive research that includes subjects from a wide variety of cultural backgrounds. And, most importantly, an integral component of trauma research as well as trauma therapy and trauma-informed policy is to challenge cultural taboos and cultural rules. From a mental health as well as a human rights perspective, it is necessary to address sexual violence even in traditional Catholic and Muslim societies, and it is possible and necessary to challenge the practice of physical punishment of children in Africa. Culture is an important factor that has to be considered in treatment and programming, but culture is not always right (see also William Yule's contribution further down).

## Misari Oe, Kurume University, Japan: verbalization, societal stigma and self-stigma

A prevalence study of trauma and PTSD in Japan (Kawakami, Tsuchiya, Umeda, Koenen, & Kessler, 2014) revealed that approximately 60% of the respondents reported “exposure to at least one traumatic event in their lifetime.” Interestingly, 15% of these respondents did not describe the contents of their traumatic events but rather reported them as “private events” or “some other event.” This occurrence was higher than that in other countries such as Australia and South Africa. More importantly, this group showed a higher conditional risk of PTSD. This means that they might conceal their severe traumatic experiences such as rape because of embarrassment or stigma.

In clinical practice, some patients want their therapists to “guess the cause of their complaints WITHOUT describing the contents of trauma or their stressful life events,” or “cure WITHOUT clarifying the causal relationship between stressors and symptoms.” Although cognitive behavioral therapies (CBTs) are available to some extent and are also suitable for some patients, it is also true that there are many patients who despise rationale-driven psychotherapies. If therapists would apply CBT to these patients, it is likely that they would leave their therapists in silence without any complaint. Trauma narratives could appear after a patient–therapist relationship is sufficiently established, and yet, some patients seem extremely sensitive about verbalizing what has happened in their lives. For instance, a patient told me that she was so nervous that she felt the “pressure of confrontation” even when she woke up on the very day of her scheduled therapy session. Thus, “verbalize or not” has a great impact in psychotherapy in Japan.

Japanese society is rather more collectivistic than individualistic. In collectivistic cultures, harmony within the group is of highest priority (Hofmann, Asnaani, & Hinton, 2010). Conversely, the public tends to exclude people categorized as “outside of social norms.” This kind of stigmatization often happens after the occurrence of personal traumatic events in peoples’ lives. For example, Maeda and Oe (2015) pointed out that self-stigma was one of the characteristic psychological impacts after the 2011 Fukushima Daiichi Nuclear Power Plant accident. The young survivors concealed that they were residents of Fukushima and some survivors even believed that they were not allowed to marry or have children, inducing the public stigma related to radioactive contamination. This discrimination is similar to the current trend of a culture of bullying at school; and, indeed, while looking at Japan in its entirety, many Japanese students have committed suicide showing the somewhat dark side of an otherwise harmonized society.

## William Yule, King's College, London, UK: the abuse of “culture”

When we began presenting empirical findings that showed that an evidence-based group approach based on CBT principles helped children in a number of very different countries to gain better control of their stress reactions, we were often met with a mantra-like reaction: “Aren't you just imposing western-based therapy on other cultures?” Such a Pavlovian-style reaction needs unpicking. The comments often came from very well-meaning humanitarian workers who had good reason to question whether all aspects of Western exports were benign. There is still a lot of guilt and anger focused on colonialism of the 19th century. In addition, there is a distrust of “the medical model” as if there were only one such thing. Along with this is the muddled view that “labelling is wrong”—without being clear that labelling and diagnosis is a way of communicating about reactions, NOT about disrespecting the individual. Identifying problems can lead to better ways of helping.

Surely most people who want to help children (and adults) affected by wars, disasters, and personal crises accept that the resulting stress reactions come about as a result of an interplay between biological and psychosocial factors. While the early studies on PTSD were valuable in drawing attention to a triad of responses that seemed to identify a near-universal reaction to major threats, subsequent experience has shown that the situation is much more fluid. It is true that in nearly all cultures where it has been examined, the broad reactions are remarkably similar. Human beings show more similarities than differences in their responses to threat. But equally, being brought up in different cultures with different traditions, the ways in which some of the reactions are expressed may differ. Some constructs may not be expressed in single words; some may be expressed in what may seem to be roundabout language. But it is still the case that anxiety, depression, and stress reactions are universally present.

For many years, adults—parents and teachers alike—avoided seeing major stress reactions in children. In large part, they were scared to talk with children in case it marked the child for life. Now we know that children benefit from talking about their reactions—not being forced to talk; but being listened to sympathetically. The days of staff in international humanitarian organizations refusing to acknowledge that a child could be traumatized are surely over (especially as mentioning child trauma can be a great fund raiser!).

We still have a great deal to learn about how best to help children affected by a traumatic event. The evidence is accruing that it is indeed best to begin help as early as possible. “Watchful waiting” and DSM-style 1- to 6-week inactivity are not supported. Most agree that survivors should not be forced to talk of their experiences and that single one-off attempts at intervention are a travesty to be avoided. Equally, whatever help is offered should be acceptable within the community. But one has to start somewhere and where better than by sensitively adapting intervention techniques that have been demonstrated to help others in similar situations. All interventions should be carefully monitored in any case, and if additional interventions are suggested from different cultures, they too should be properly evaluated. It is not enough to say that “traditional healing” should be followed without hard evidence that that is helpful and not harmful.

Fourteen years ago, Dyregrov and collaborators asked “Is the culture always right?” (Dyregrov, Gupta, Gjestad, & Raundalen, 2002). Fourteen years on, the world is still finding it difficult to confront some traditional practices such as physical punishment of children or female genital mutilation. Culture has to be considered alongside biology, and both need to be rigorously tested.

## Richard A. Bryant, University of New South Wales, Sydney, Australia: the issue of resources

One important aspect of treating PTSD and other trauma-related conditions in different cultural contexts is the recognition of our limited evidence base. The significant majority of the global burden of disease arising from mental health conditions occurs in low and middle income countries (LMICs) (Ferrari et al., 2013). These mental health problems frequently arise as a result of traumatic events, including war, mass violence, natural disasters, and accidents. They are further compounded by poverty, overcrowding, and general hardship. In this context, it has been repeatedly noted that the social costs of mental health problems are markedly higher in LMICs than in developed nations (Patel, 2007). Despite this global pattern, relative to our considerable knowledge on how to treat mental health conditions in Western contexts, we know much less about how to treat these conditions in developing countries. Reflecting this situation, over 80% of published research on mental health comes from developed nations; in contrast, only 6% of this research comes from LMICs (Saxena, Paraje, Sharan, Karam, & Sadana, 2006). More specifically, in the field of traumatic stress research, 13% of studies are performed in LMICs (Fodor et al., 2014).

One of the core issues to be considered in treating posttraumatic stress conditions globally is the limited resources available to LMICs. There is enormous inequity in distribution of health resources worldwide. For example, there are more psychiatrists in the United States than in the two most populated countries on the planet (India and China) and the entire African continent combined (Patel & Thornicroft, 2009). It is unsurprising that over 90% of people with a mental disorder in LMICs are untreated (Wang et al., 2007). This raises a serious challenge for evidence-based treatments that we use for PTSD and other conditions, because they are typically resource-intensive, rely on reasonably qualified people to deliver them, and presume a basic health infrastructure in which they can be delivered. Most LMICs do not have these resources available to them, and hence, it is difficult for them to implement services that are akin to what is currently delivered in developed nations. When one peruses the literature, we see that evidence-based treatments (e.g., trauma-focused psychotherapy) have been successfully delivered in LMICs (Bolton et al., 2014). However, these examples are rare and the unfortunate pattern is that once a research trial is complete, one typically does not see the implementation of the intervention being conducted in a systematic way in the LMIC in which the trial was conducted.

It is naïve to conclude that we can simply transpose evidence-based interventions to LMICs because, by definition, they lack the resources to permit optimal training and supervision that is required to ensure that local capacity is sustained to deliver the interventions. It is for this reason that there are initiatives underway, including by the World Health Organization (Dawson et al., 2015), to test interventions that can be trained to local providers in an affordable manner, implemented in a way that is sustainable in the context of local resources, and yet effective in terms of achieving the goals that the intervention is striving for.

## Conclusions

The aim of this article was to describe and highlight some aspects that therapists should be aware of when treating patients who suffer from trauma-related disorders. Although we do not claim to have provided a systematic and comprehensive overview, a number of important points clearly emerge.

Given the increasing globalization of our world, we all interact on a daily basis with people from cultural backgrounds different from ours, and this is true for both our private and our professional lives. The “refugee crisis” Europe is currently struggling with (Alisic & Letschert, 2016; Turner, 2015) is just one of many manifestations of this development. In response to this reality, psychotherapists working with trauma survivors need to further develop their cultural competence and sensitivity.

When working in a “Western” setting with patients from diverse cultural backgrounds, it may be helpful to reflect on rigidly held cultural beliefs such as the view that rape victims bring shame on their families. Involving people from the patient's same cultural background who have a more nuanced view may provide the therapist with a more accurate appraisal and help the patient find a way out of what initially looks like a dead end. Some patients are reluctant to talk to their therapists about their traumatic experiences, which is a sine qua non in virtually all evidence-based trauma-focused treatments. However, they may be willing to write or use other ways of accessing the painful memories such as drawing, painting, dancing, singing, or playing an instrument. Working with physical symptoms connected to the trauma or visiting places that evoke memories may also be useful “detours” or alternatives, preparing the patient to talk directly about the trauma. Aram Hasan's case vignette is a nice demonstration of how the therapist's suggestion to use his patient's drawings helped to unblock the therapeutic process.

The individual and collective meanings linked to trauma and trauma-related disorders vary across cultures: while in the individualistic “Western” world, therapists are used to survivors’ feelings of being vulnerable or inadequate, in more collectivistic cultures such as Japan, appraisals about social functioning or evaluation by others are much more common. Misari Oe provides us with an impressive and initially counter-intuitive example, namely the additional burden of stigma and self-stigma the survivors of the 2011 Fukushima Daiichi Nuclear Power Plant accident were, and still are, confronted with over and above the physical and psychological stressors they had had to endure. And Gladys Mwiti's case vignette demonstrated the importance of community and family cohesion in her patient's struggle to recover from the psychological impact of traumatic bereavement.

Empirically supported psychotherapies for PTSD can be successfully implemented across cultures (Acarturk et al., 2015). However, a number of challenges need to be taken into account: many LMICs have very limited resources available overall, and more specifically, they suffer from a poor health infrastructure. Furthermore, as we learned from Christian Kristensen, in countries where highly prevalent violence is combined with a tradition of impunity, patients may be absolutely correct in perceiving their environment as entirely dangerous. In such an environment, therapeutic efforts are compromised because trauma survivors will have to deal with justified feelings of helplessness or anger, associated with ruminations about injustices or fantasies of revenge.

Finally, culture is not always right (Dyregrov et al., 2002). We need to beware of premature cultural stereotyping and false dichotomies such as Western versus non-Western cultures or developed countries versus LMICs. We should not abuse “culture” as an excuse for not providing effective treatments. We treat persons not disorders. Thus, culture-sensitive psychotraumatology means assuming an empathic and non-judgmental attitude, trying to understand each individual's cultural background. If we meet our patients with a genuine interest in their unique system of beliefs and attitudes, we can learn a lot from them. Thus, developing cultural sensitivity will enrich our work and provide us with opportunities to learn how diverse human beings are.
